# A high-resolution hydrodynamics and sediment dataset in the coastline waters of the Northern Bay of Bengal

**DOI:** 10.1016/j.dib.2024.110577

**Published:** 2024-06-01

**Authors:** Mohd Imaran, Arun Chakraborty, Subhasish Tripathy

**Affiliations:** a Centre for Ocean, River, Atmosphere and Land Sciences (CORAL), Indian Institute of Technology Kharagpur 721302, India; b Department of Geology and Geophysics (GG), Indian Institute of Technology Kharagpur 721302, India

**Keywords:** ROMS-CSTMS, Temperature-salinity, Sand, Silt, Bottom stress

## Abstract

Regional Ocean Modeling System (ROMS) - Community Sediment Transport Modeling System (CSTMS) model used to acquire a dataset of physical variables and sediment on the continental shelf of India and countries adjacent to the Northern Bay of Bengal. The high-resolution model resolved the complex bathymetry taken from ETOPO2, forced by COADS climatological winds. Furthermore, the tides are taken from TPXO7 and lateral boundaries from SODA with initial condition from WOA09 and sediment concentration set to zero in the simulation. The river discharge as the point source of nine rivers, which are in our domain was obtained from Dai and Trenberth climatology. The raw data of two alternative simulations, with river and without river was obtained. The Large-McWilliams-Doney (LMD) vertical mixing scheme was used in both scenarios for the simulations. The output data was stored in the NetCDF format for 12 months with daily data availability of two different cases: with river and without river simulation. This dataset can be accessed from the following link https://www.scidb.cn/en/detail?dataSetId=0218045285984221bef97b0834c5eaf4.

Specifications Table

**Table:** 

Subject	Oceanography
Specific subject area	Coastal Hydrodynamics and Physical Oceanography
Type of data	NetCDF files
Data collection	The data were obtained from the simulation of ROMS-CSTMS hydrodynamic modeling
Data source location	Country: India Latitude and Longitude: Indian Ocean Modeling Laboratory (IMOD) Centre for Ocean, River, Atmosphere and Land Sciences (CORAL), Indian Institute of Technology Kharagpur (22.3149° N, 87.3105° E) GPS coordinates: N 22°19${}^{\prime }$, E 87°18${}^{\prime }$ Collected samples/data: For specific region 15° N - 26° N, 80° E- 98° E
Data accessibility	Repository name: Science Data Bank Data identification number: https://doi.org/10.57760/sciencedb.17226 Direct URL to data: https://www.scidb.cn/en/detail?dataSetId=0218045285984221bef97b0834c5eaf4

## 1 Value of the Data

• The raw data contains the numerical simulations of the hydrodynamics in the coastal zone of India and adjacent countries, which can be utilized by various physical oceanographers studying the nature of ocean currents, the movement of sediment particles under the combined action of waves and currents alongside Indian east coast and coastal waters of the Bay of Bengal.
• Biogeochemical oceanographers can also use these datasets to examine how physical processes can affect the value of pH, sediment rich nutrient supply, net primary production, and loss of marine life due to high siltation in the coastal zone and aquatic habitat.
• Deltas are the depositional zones where substantial volumes of sediment are held in reserve because of stream power loss, bifurcation, and the presence of various wetlands. Sediment dataset can also be useful in the progradation, and aggradation of the delta system. The Ganges-Brahmaputra-Meghna collectively formed a massive delta system known as the GBM delta. This modeling approach can help to understand the dynamics of sediment particles under an estuarine environment.

## 2 Background

The Ganges-Brahmaputra River, which ranks first among all rivers in the world alongside the Amazon, currently delivers 1×10^9^ tonnes/year of sediment load to the head of the Bay of Bengal [1]. Tides over the Bay of Bengal have a dynamic relevance as suggested by Bhagawati et al. [2], which caused a monthly drop in the depth of Mixed Layer across the Bay of Bengal. Freshening shown by the northern Bay is due to the dispersal of the river plume, which influences seasonal stratification as the barrier layer forms and freshwater is subsequently drawn in by fronts and eddies alongside the East India Coastal Current (EICC) [3]. Research by [4] reveals the current pattern along the Indian coast at depths of 100, 500, and 1000 m. A Multiscale Modeling and Forecast System with High Resolution was developed by Chakraborty and Gangopadhyay [5,6] for the Bay of Bengal which is Based on climatological simulation. To replicate the impact of the dynamics of tides on biogeochemistry in the Hooghly Estuary by [7] exhibits the highest levels of chlorophyll-a productivity in the suspended sediment areas with severe bottom stress caused by tidal forces and coastal currents. Nevertheless, prior research has not included modeling studies of sediment transport patterns along the interaction of river discharge with waves and ocean currents. Since very little information is available about the dynamics of sediment with ocean currents and waves around the Indian shores, the dataset might be helpful to a variety of scholars.

## 3 Data Description

**Fig. 1:**
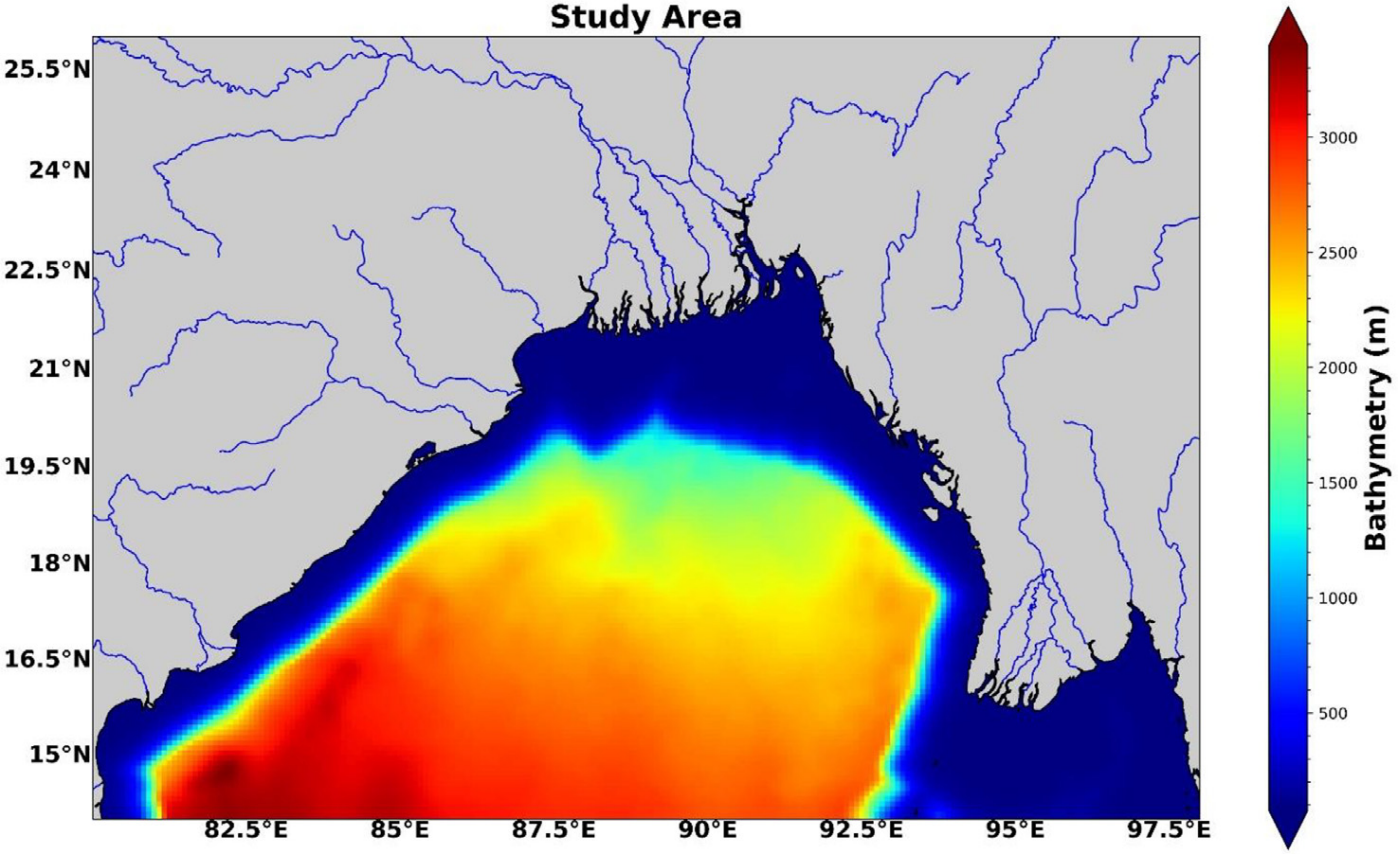
Study region (15° N - 26° N, 80° E- 98° E) along with colored shaded bathymetry (m).

The high-resolution dataset of sediment in the Coastal waters of the Northern Bay of Bengal is an output of ROMS-CSTMS modeling for the ten years climatological run. The study region (Fig. 1) is located at 15° N - 26° N, 80° E- 98° E, and The dataset contains the daily mean of hydrodynamic parameters, sediment particles (fine sand and silt),and temperature-salinity structure. Two simulations were performed which are **with river** and **without river** and the dataset was saved in their respective directories. There are 24 files in the dataset, 12 files in each directory. Every output file is named under the standard HIS and AVG files obtained from the ROMS-CSTMS modeling. The average and history files are named **“roms_avg_*Y*<Month_number>.nc”** and **“roms_his_*Y*<Month_number>.nc”**, respectively. where Month_number ranges from 1 to 12 and each month is considered to have 30 days. The dataset was saved in NetCDF format after the simulation of each month was completed. The ROMS-CSTMS model was setup at 1/12^°^ of horizontal resolution covering approximately 9 km horizontal distance, with 32 vertical levels for the study region located at 15° N - 26° N and 80° E - 98° E.

**Fig. 2:**
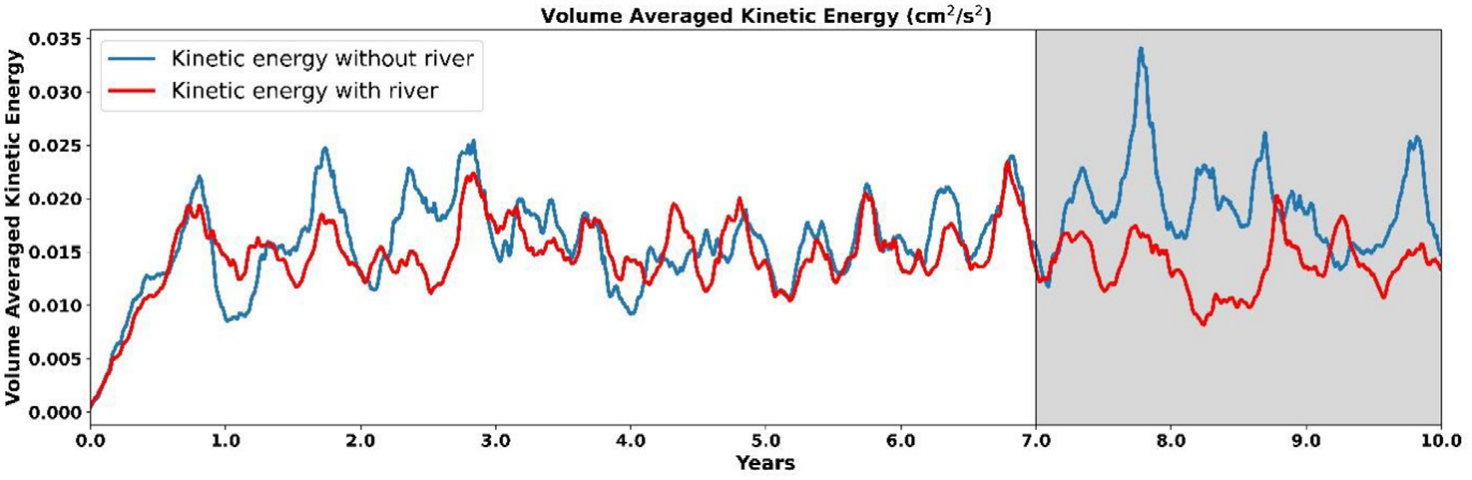
Volume averaged kinetic energy obtained from ROMS-CSTMS with and without river simulations.

Both simulations have been run for ten years, as was previously specified, the model achieved the annual cycle within three years and reached the almost a steady annual cycle state from seventh year (Fig. 2). We have used modeled climatological data from the last three years of the simulations and presented here (Fig. 2). Numerous simulations were completed for our future research based on these simulations at low resolution (1/4°) to assess the effects of the domain extension besides other background parameters such as the slope parameter **``r''** that is influenced by topography, the Laplacian diffusivity number on a horizontal scale, and the coefficient of bottom drag.

**Fig. 3:**
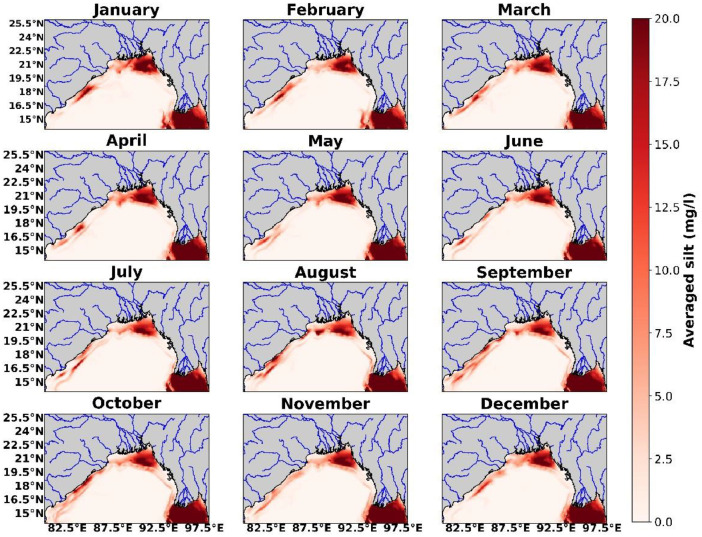
Silt concentration near the mouth of major delta system obtained from ROMS-CSTMS climatological simulation with the river.

**Fig. 4:**
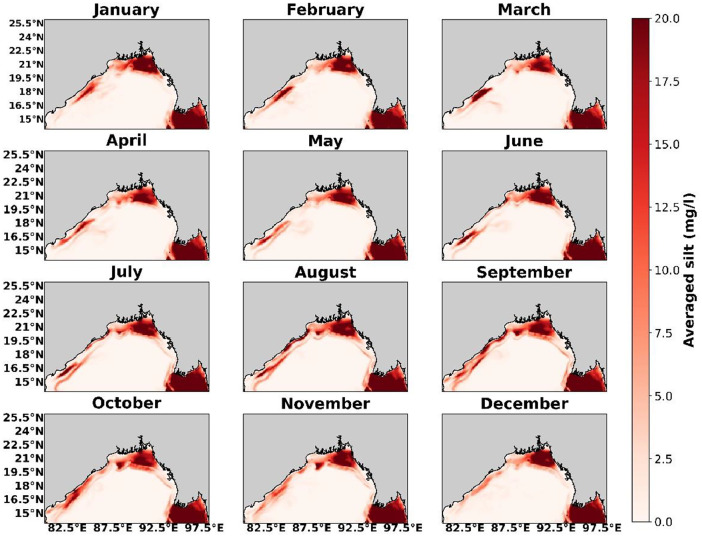
Silt concentration near the mouth of major delta system obtained from ROMS-CSTMS climatological simulation without the river.

This made it possible to evaluate the model's sensitivity to small changes in its starting parameters and then discuss which configuration is optimal for simulations with increased resolution. Monthly climatology of the Silt of the domain obtained from ROMS-CSTMS with river and without river simulation at 1/12° resolutions are shown in Fig. 3, Fig. 4.

The ROMS AGRIF model has been written in FORTRAN and is freely accessible at https://data-croco.ifremer.fr/CODE_ARCHIVE/Roms_Agrif/Roms_Agrif_v3.1.1_07_07_2014.tar.gz for usage in numerical simulations of coastal hydrodynamics and hydro-thermodynamics [8,9]. To start with the model simulation, surface forcing as input files, discharge from rivers, and for initial and boundary conditions, the **ROMSTOOLS** is utilized which is produced by Penven et al. [10]. A collection of scripts in **ROMSTOOLS** is included with the model package and is available to work in **MATLAB.** Additionally, **ROMSTOOLS** includes several scripts that enable MATLAB to visualize the initial and modeled outputs. Furthermore, **ROMSTOOLS** is open to download as a whole package at https://data-croco.ifremer.fr/UTILITIES/Utilities_ROMSTOOLS_v3.0_21_12_2012.tar.gz.

The current components have a different coordinate system than the other features in the coastal waters of the northern Bay of Bengal. The latitude and longitude of the zonal components of the ocean currents are represented by the variables **lat_u** and **lon_u**, respectively (Table 1) and The latitude and longitude of the meridional components are denoted by the variables **lat_v** and **lon_v** of ocean current, respectively. For the remaining variables **lon_rho** and **lat_rho** are used for longitudes and latitudes, respectively.

**Table 1:** Hydro-thermodynamic parameters and sediment particles obtained from ROMS-CSTMS modeling.

Variables	Description	Unit
lon_rho	longitude at rho points	°E
lat_rho	latitude at rho points	°N
lon_u	longitude in zonal direction	°E
Lat_u	latitude in zonal direction	°N
lon_v	longitude in meridional direction	°E
Lat_v	latitude in meridional direction	°N
h	Depth of the Ocean	m
mask_rho	Land mask (0 for land, 1 for water)	–
temp	P otential temperature	°C
salt	Salinity	PSU
sand	Averaged sand sediment	mg/l
silt	Averaged silt sediment	mg/l
u	momentum towards the east	m/s
v	momentum towards the north	m/s
w	momentum in Vertical direction	m/s
zeta	Sea surface height	m
ubar	u-momentum component integrated in vertical	m/s
vbar	v-momentum component integrated in vertical	m/s
f	The Coriolis term	s^−1^
bostr	Kinematic stress at the bottom	N/m^2^
wstr	Kinematic stress caused by wind	N/m^2^
sustr	Kinematic component of wind stress (u)	N/m^2^
svstr	Kinematic component of wind stress (v)	N/m^2^
Diff3d	Coefficient of average horizontal diffusion	–
AKt	Coefficient of average temperature vertical diffusion	m^2^/s
hbl	The planetary boundary layer depth	m
hbbl	The bottom boundary layer depth	m
shflux	Net heat flux at the surface	W/m^2^
swflux	Freshwater flux at the surface (E-P)	Cm/day
swrad	Surface radiation in the short wave	W/m^2^
bed_thick	Averaged thickness of sediment bed layer	m
bed_poros	Averaged porosity of sediment bed layer	–
bed_frac_sand	Averaged volume fraction of sand in bed layer	–
bed_frac_silt	Averaged volume fraction of silt in bed layer	–

The depth of ocean/bathymetry (**h**), mask (**mask_rho**), and Coriolis parameter (**f**) are the only two-dimensional parameters. Mask is a binary factor and is utilized to demarcate the landmass from the ocean. It has the value of 1 and 0 for water and land, respectively. The remaining parameters, which consist of three or four dimensions, are explained in Table 1. Each hydro-thermodynamic parameter has four dimensions, except for the sea surface height (**zeta**), which has three dimensions as its dependence is limited to time and geographic location.

## 4 Experimental Design, Materials and Methods

The community sediment transport model is a subroutine of the ROMS model which is used in this study to acquire the dataset. ROMS is a free-surface with extended, vertical s-coordinates which are terrain-following and horizontal coordinates that are orthogonally curvilinear, hydrostatic, primitive equation model [8,9]. The early sediment-transport capability in ROMS focused on the effects of various turbulence closure schemes on sediment flow. To take into account, the impact of mixed beds on the entrainment of sediment, a two-layer sediment bed has been considered which is similar to [11] in the modeling. Furthermore, the combined influence of currents and waves on the bottom stress and also the corresponding Surface wave and bottom ripple parameterization have been presented. where the diameter is represented by d, $\rho_{s}$ the sediment grain density, $w_{s}$ is the settling velocity, $E_{u}$ is the erosion rate parameter, and $\tau_{cr}$ the critical shear stress. The initial substrate thickness is fixed at 1 m, whereas the active layer thickness is $\delta_{a}=3\;mm$, and the porosity, is p=0.4. Table 2 contains the model input parameters. For the two experiments that are **with river** and **without river,** ripple length and ripple height set to zero initially. Additionally, in the simulation, the initial concentration of sediment particles also set to zero, with their respective fractions

(1) $$\left(f_{silt},f_{sand}=1-f_{silt}\right)$$

Eq. (1), local depth-based analytical function) corresponding to a depth of 10 m [12]. Grain size is empirically correlated with entrainment rate, settling velocity, and critical shear stress. The empirical relationship of grain size is used to calculate settling velocity [13].

**Table 2:** Two classes of sediment and their parameters for climatological run.

Class name	*d (µm)*	$\rho_{s}$_(kg/m_^3^_)_	$w_{s}$_(mm/s)_	$E_{u}$	$\tau_{cr}$ (N/m^2^)
Silt	24	2650	0.4	1.0 $\times \;10^{-4}$	0.07
Sand	125	2650	9.4	2.5 $\times \;10^{-3}$	0.15

A large, multinational community has been using ROMS in major parts of the world's oceans. It also provides the ability to use Lagrangian floating particles and passive tracers in a particular experiment. Additionally, modules from disciplines that rely on hydrodynamics, such as sedimentology, biogeochemistry, and the interplay of waves and currents, can be included. For the current numerical experiments, we used the version of ROMS created at the “Institut de Recherche pour le Développement” (IRD) by Patrick Marchesiello (IRD Toulouse), in collaboration with Pierrick Penven, Gildas Cambon (IRD Brest), and Laurant Debreu (INRIA, Institut National de Recherche en Informatique et en Automatique). A user can easily generate the initial conditions of a simulation and analyze or view the output data within the Matlab environment by implementing the ROMSTOOLS package [10]. In addition, this version of ROMS supports the usage of the AGRIF library (Adaptive Grid Refinement in Fortran), which permits nesting of different grids one within another, increasing their unique resolutions [14].

Particularly, modelers have already made extensive use of ROMS aimed at the Bay of Bengal [2,3,5,6,[15], [16], [17]]. One important advantage of ROMS is that it was created to operate at regional scales inside a framework that is both idealised and practical. Essentially, this model makes it simple to set up and do sensitivity runs by varying a wide range of factors, which enables the realisation of process-oriented modeling.

We have tested the impact of river flow on sediment dynamics concerning waves and currents by obtaining datasets from two alternative scenarios: 1) with river and 2) without river simulation using ROMS-CSTMS. We used a geographical domain of the Northern Bay of Bengal, which included the entire East Coast of India and the Bengal coast, the Southeast Coast of Bangladesh, and the Southwest Coast of Myanmar, in both simulations. We have utilized a horizontal resolution of 1/12° (∼ 9 km), The sediment subroutine module has 2 layers and 32 vertical levels are intended to depict the physical processes for the water column realistically.

In reality, to obtain a higher resolution in the upper layers, ROMS has a default tendency to extend these vertical levels toward the ocean's surface. To obtain additional subsurface resolution at certain depths of concern, it is also feasible to create an extra uniform division of the vertical levels. For instance, the decision to choose the latter was motivated by our desire to learn more about sediment dynamics with river discharge, which leads to mixing, breaking, and dissipation. To assess both the initial model modification and the spin-up time needed to bring the simulated energy budget to a statically steady state, these simulations were run for ten years, saving the result daily.

The classical variables that have an Eulerian analysis (i.e., potential vorticity, kinetic energy, water mass characteristics and volumes, heat, and salt fluxes) were used to investigate the development of this equilibrium. Additionally, we calculate the required amount to test the model's sensitivity to additional initial parameters: surface fluxes from climatology and wind forcing; fluctuating slope parameter values that regulate the inclination of the slope of the continent and the smoothness of bottom topography; for an idealized water column; subsequently activation of a diffusive element of advection scheme, which is intended to lessen the synthetic results of fictitious diffusional mixing that might arise within the fields for tracers (Temperature and Salinity).

The grid file was created using the datasets and circumstances listed below, forcing in both the starting state and the open boundaries the **“River flow”** and **“No-river flow”** configurations for comparability of two distinct simulations.

• ETOPO2 data is used to derive bathymetry, which is then smoothed using the condition that;
  (2) $$\frac{\Delta h}{2h}<0.2$$
• With a bottom depth of 10 m;
• Sources of heat, wind forcing, and freshwater are taken from the Comprehensive Ocean Atmosphere Dataset (COADS) for the surface climatology;
• The Simple Ocean Data Assimilation dataset (SODA) V-2.1.6 is used to enforce an active, implicit, the lateral open boundaries with an upstream biased radiation condition, which links the model to the surrounding ocean;
• The climatology of largest Asian rivers’ discharge [18], which comprises monthly means values of runoff, has been employed;
• The TPXO7 package [19] provided the 10 tidal components (diurnal and semidiurnal), which included altimetry data from many satellites to verify the hydrodynamic model's results;
• A few well-data points in the world's ocean have regional climatology for salinity and temperature at 1/10° In honor of Sydney Levitus, the WOA is also referred to as “Levitus” or the “Levitus Climatology” [20], its Pioneering founder;
• The Laplacian and biharmonic linear combination, scaled with the grid size, is the parametrized form for the horizontal mixing. The **KPP parametrization** is the basis of the vertical mixing (**LMD** mixing scheme) [21].

## Limitations

In this high-resolution modeling, we are dealing with silt and fine sand only with hydro-thermodynamical variables. However, coarser sand is out of the scope of the dataset.

## Ethics Statement

The authors have confirmed that the current study does not contain animal trials, human beings, or any data gathered from social media sites. They have also reviewed and complied with the ethical standards for publication in Data in Brief.

## CRediT authorship contribution statement

**Mohd Imaran:** Conceptualization, Methodology, Validation, Formal analysis, Investigation, Writing – original draft, Visualization. **Arun Chakraborty:** Conceptualization, Validation, Writing – review & editing, Supervision. **Subhasish Tripathy:** Conceptualization, Validation, Writing – review & editing, Supervision.

## Data Availability

High resolution modeling of sediment in the coastal waters of the Northern Bay of Bengal (Original data) (Science Data Bank)
